# A predictive nomogram for assessing the likelihood of retrieving 12 lymph nodes after rectal cancer surgery: a single-center study

**DOI:** 10.3389/fonc.2025.1617058

**Published:** 2025-08-25

**Authors:** Jian Ma, Runyang Hao, Shuai Jiao, Qingmin Chen, Baohong Yang, Xu Guan, Jiale Li, Xinxuan Zhao, Yu Huo, Qingxia Xu, Haiyi Liu, Wen Su, Xishan Wang

**Affiliations:** ^1^ Department of Colorectal Surgery, National Cancer Center/National Clinical Research Center for Cancer/Cancer Hospital, Chinese Academy of Medical Sciences and Peking Union Medical College, Beijing, China; ^2^ Department of Colorectal Surgery, Shanxi Province Cancer Hospital/Shanxi Hospital Affiliated to Cancer Hospital, Chinese Academy of Medical Sciences/Cancer Hospital Affiliated to Shanxi Medical University, Taiyuan, China

**Keywords:** rectal cancer, lymph node, nomogram, CA19-9, hemoglobin

## Abstract

**Objective:**

The retrieval of 12 lymph nodes (LNs) remains a crucial criterion for accurate staging and prognosis evaluation in rectal cancer (RC). However, some patients fail to meet this threshold after surgery. This study developed a nomogram model based on clinical variables to predict the probability of retrieving 12 LNs postoperatively.

**Methods:**

Patients who underwent radical RC surgery at Shanxi Cancer Hospital between 2015 and 2020 were retrospectively analyzed. Continuous variables were converted into categorical variables. Chi-square tests were used to identify key factors influencing the retrieval of 12 LNs. Significant variables were incorporated into a nomogram model. The model’s discrimination ability was evaluated based on the receiver operating characteristic (ROC) curve, while model calibration was assessed using calibration plots. The clinical utility of the model was determined using decision curve analysis (DCA).

**Results:**

A total of 2,724 RC patients were included; 1,906 cases were assigned to the training dataset, while 818 were assigned to the internal validation dataset. Chi-square analysis identified age, T stage, N stage, tumor size, Carcinoembryonic Antigen, CA19-9, hemoglobin, and platelet count as significant factors associated with 12 LN retrieval. The nomogram indicated that T stage, N stage, and tumor size contributed most significantly. The areas under the ROC curves of the model were 0.669 for the training dataset and 0.689 for the internal validation dataset. The calibration plots showed good agreement between the predicted probabilities and actual outcomes. The DCA curves demonstrated a favorable net benefit across a wide range of threshold probabilities.

**Conclusion:**

The nomogram model can effectively predict the likelihood of retrieving 12 LNs following RC surgery. The model also provides a valuable tool for preoperative risk stratification and personalized clinical decision-making.

## 1 Introduction

Rectal cancer (RC) is among the most common malignancies of the digestive system, and its incidence and mortality rate are increasing worldwide (1). Surgical resection remains the primary treatment modality, and inadequate resection margins may contribute to disease recurrence (2). Pathological examination is still considered the gold standard for evaluating RC staging, and insufficient lymph node (LN) retrieval may compromise the accuracy of tumor staging (3). Multiple clinical guidelines recommend the retrieval of at least 12 LNs following surgery to ensure accurate staging and guide subsequent treatment decisions (4, 5).

Despite the widespread acceptance of this “12 LNs” standard, a considerable proportion of RC patients fail to meet this threshold in clinical practice (6). The number of LNs retrieved is influenced by a variety of factors including the surgical technique, pathological process, and patient-specific clinical characteristics (7–10). Although previous studies have explored the relationship between clinical variables and LN yield, comprehensive investigations specifically targeting RC patients remain limited.

The accurate identification of clinical factors affecting LN retrieval is crucial for both preoperative assessment and postoperative management (11–13). This information can assist surgeons in formulating more precise intraoperative strategies to maximize LN harvest; it may also help identify high-risk patients postoperatively and provide a basis for further therapeutic interventions. In this study, based on a through retrospective analysis of multiple clinical variables, we developed a simple and effective predictive tool to estimate the probability of retrieving 12 LNs in RC patients after surgery. This predictive model offers valuable insights for clinical evaluation and individualized treatment planning.

## 2 Methods

### 2.1 Patients

The patients included in this study were treated at Shanxi Cancer Hospital between 2015 and 2020. Baseline clinical information and pathological results were carefully verified for accuracy. The serum levels of carcinoembryonic antigen (CEA), carbohydrate antigen (CA19-9), and routine blood parameters were measured using standardized equipment to ensure data consistency. All samples were collected as fasting venous blood on the second day after hospital admission. Neutrophils, lymphocytes, hemoglobin (Hb), and platelet count (PLT) were categorized into three groups based on the reference ranges provided in the corresponding routine blood test reports. Strict inclusion and exclusion criteria were applied to enhance the reliability and validity of the results.

Inclusion criteria: Patients diagnosed with RC who underwent radical surgical treatment.

**Figure 1:**
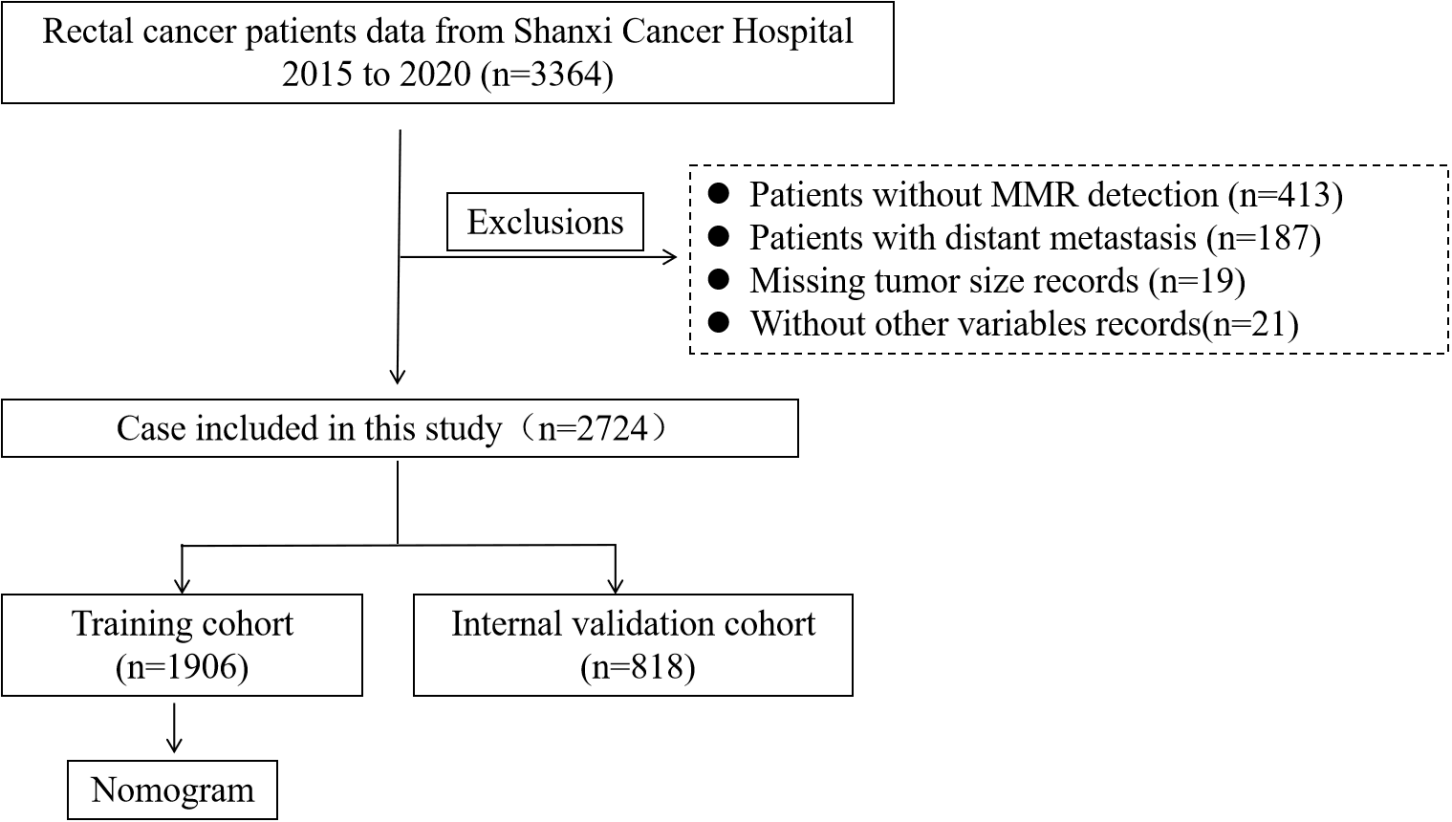
Flowchart of the RC patient’s selection from Shanxi Cancer Hospital, 2015-2020. (RC, rectal cancer).

Exclusion criteria: (1) Missing data of mismatch repair information; (2) Presence of distant metastasis; (3) Missing data on tumor size; (4) Incomplete information on other variables (**Figure 1**).

### 2.2 Variable processing and nomogram construction

The T and N stages were classified according to the 8th edition of the AJCC staging system. MMR status and tumor size were determined by pathologists. BMI was categorized into four groups according to the World Health Organization classification. Tumor size was divided into >=4 and <4 cm based on the median value. CEA and CA19–9 were grouped using their respective normal reference ranges. Blood parameters including neutrophils, lymphocytes, hemoglobin (Hb), and platelet count (PLT) were categorized into three groups: below normal, within the normal range, and above normal. LN count was dichotomized into two groups: < 12 and ≥ 12.

All eligible patients were randomly assigned to the training dataset or the internal validation dataset in a 7:3 ratio. The nomogram model was developed using the training dataset and incorporated the following variables: age, T stage, N stage, tumor size, CEA, CA19-9, Hb, and PLT.

### 2.3 Statistical analysis

Categorical variables were presented as frequencies and percentages, and comparisons between groups were performed using the Pearson chi-square test. A nomogram is a widely used visualization tool. In this study, the nomogram was constructed to predict the probability of retrieving 12 LNs based on the selected variables. The predictive performance of the model was evaluated based on the receiver operating characteristic (ROC) curve. Decision curve analysis (DCA) was conducted to assess the clinical utility of the nomogram by quantifying the net benefits across a range of threshold probabilities.

All statistical analyses were performed using R software (version 4.3.2). The rms, pROC, ggDCA, and ggplot2 packages were used to construct the nomogram and plot the ROC curves, DCA curves, and calibration plots. A two-sided *P*-value < 0.05 was considered statistically significant.

## 3 Results

### 3.1 Patient characteristics

A total of 2,724 patients diagnosed with RC were enrolled in this study based on predefined inclusion and exclusion criteria. These patients were randomly assigned to a training cohort (n = 1,906) and an internal validation cohort (n = 818) using a 7:3 ratio to ensure a balanced distribution for model development and validation. Among all patients, 1,601 were male (58.74%) and 1,605 (58.96%) were aged over 60 years. Regarding tumor characteristics, the majority of patients presented with advanced local disease. Specifically, 51.91% had T3 stage tumors, and 58.85% had no lymph node metastasis (N0 stage). Additionally, most tumors were relatively large, with 62.92% measuring ≥ 4 cm in diameter. In terms of preoperative serum markers and hematological parameters, the levels of CEA, CA19-9, neutrophils, lymphocytes, Hb, and PLT were within their respective normal reference ranges in the majority of patients, reflecting relatively stable systemic conditions at the time of admission. Of particular note, 826 patients (30.36%) had fewer than 12 LNs retrieved during surgical resection, which may impact accurate pathological staging and subsequent treatment decisions. The detailed baseline characteristics of the patients are summarized in **Table 1**.

**Table 1:** Demographic and clinicopathological characteristics of the training and internal validation datasets.

Variables	Training dataset N=1906 (%)	Internal validation dataset N=818 (%)	Total N=2724 (%)
Sex			
Males	1142 (59.92%)	458 (55.99%)	1600 (58.74%)
Females	764 (40.08%)	360 (44.01%)	1124 (41.26%)
Age (years)			
<60	774 (40.61%)	344 (42.05%)	1118 (41.04%)
>=60	1132 (59.39%)	474 (57.95%)	1606 (58.96%)
BMI (kg/m^2^)			
<=18.5	105 (5.51%)	39 (4.77%)	144 (5.29%)
18.5-24.9	1127 (59.13%)	490 (59.90%)	1617 (59.36%)
25-29.9	599 (31.43%)	267 (32.64%)	866 (31.79%)
>=30	75 (3.93%)	22 (2.69%)	97 (3.56%)
T-stage			
T1	49 (2.57%)	16 (1.96%)	65 (2.39%)
T2	443 (23.24%)	182 (22.25%)	625 (22.94%)
T3	995 (52.20%)	419 (51.22%)	1414 (51.91%)
T4	419 (21.98%)	201 (24.57%)	620 (22.76%)
N-stage			
N0	1131 (59.34%)	472 (57.70%)	1603 (58.85%)
N1	418 (21.93%)	175 (21.39%)	593 (21.77%)
N2	357 (18.73%)	171 (20.90%)	528 (19.38%)
MMR			
dMMR	74 (3.88%)	37 (4.52%)	111 (4.07%)
pMMR	1832 (96.12%)	781 (95.48%)	2613 (95.93%)
LN			
<12	560 (29.38%)	267 (32.64%)	827 (30.36%)
>=12	1346 (70.62%)	551 (67.36%)	1897 (69.64%)
Tumor size (cm)			
<4	707 (37.09%)	303 (37.04%)	1010 (37.08%)
>=4	1199 (62.91%)	515 (62.96%)	1714 (62.92%)
CEA (ug/L)			
<3	1129 (59.23%)	487 (59.54%)	1616 (59.32%)
>=3	777 (40.77%)	331 (40.46%)	1108 (40.68%)
CA19-9 (U/mL)			
<37	1229 (64.48%)	504 (61.61%)	1733 (63.62%)
>=37	677 (35.52%)	314 (38.39%)	991 (36.38%)
Neutrophil (×10^9^/L)			
<1.80	53 (2.78%)	33 (4.03%)	86 (3.16%)
1.80-6.30	1750 (91.82%)	749 (91.56%)	2499 (91.74%)
>6.30	103 (5.40%)	36 (4.40%)	139 (5.10%)
Lymphocyte (×10^9^/L)			
<1.10	124 (6.51%)	69 (8.44%)	193 (7.09%)
1.10-3.20	1710 (89.72%)	724 (88.51%)	2434 (89.35%)
>3.20	72 (3.78%)	25 (3.06%)	97 (3.56%)
Hb (g/L)			
<115	205 (10.76%)	96 (11.74%)	301 (11.05%)
115-150	1140 (59.81%)	497 (60.76%)	1637 (60.10%)
>150	561 (29.43%)	225 (27.51%)	786 (28.85%)
PLT (×10^9^/L)			
<125	49 (2.57%)	18 (2.20%)	67 (2.46%)
125-350	1673 (87.78%)	720 (88.02%)	2393 (87.85%)
>350	184 (9.65%)	80 (9.78%)	264 (9.69%)

(LN, lymph node; BMI, body mass index; dMMR, deficient mismatch repair; pMMR, proficient mismatch repair; Hb, haemoglobin; PLT, platelet.).

### 3.2 Factors associated with retrieval of 12 LNs

To identify potential factors influencing adequate LN retrieval, a Pearson chi-square test was conducted to compare clinicopathological and laboratory variables between patients with <12 and ≥12 LNs retrieved. The analysis revealed that several variables were significantly associated with 12 LN detected during surgery (**Table 2**). Specifically, age was a significant factor, with younger patients more likely to have ≥12 LNs examined. T stage and N stage also showed strong associations, suggesting that patients with more advanced local disease tended to undergo more extensive LN dissection. Tumor size was positively correlated with LN yield, with tumors ≥4 cm more often associated with ≥12 LNs retrieved. In addition, preoperative serum levels of CEA and CA19–9 were significantly related to LN retrieval status, indicating that tumor burden or biology might influence surgical or pathological outcomes. Furthermore, among hematological parameters, Hb and PLT were also significantly associated with the extent of LN retrieval, suggesting a potential link between patients’ systemic status and surgical thoroughness or nodal response.

**Table 2:** Univariate analysis based on examination of 12 lymph nodes.

LN	<12	>=12	P-value
Sex			0.485
males	494 (59.73%)	1106 (58.30%)	
females	333 (40.27%)	791 (41.70%)	
Age (years)			0.010
<60	309 (37.36%)	809 (42.65%)	
>=60	518 (62.64%)	1088 (57.35%)	
BMI (kg/m^2^)			0.121
<=18.5	42 (5.08%)	102 (5.38%)	
18.5-24.9	465 (56.23%)	1152 (60.73%)	
25-29.9	288 (34.82%)	578 (30.47%)	
>=30	32 (3.87%)	65 (3.43%)	
T-stage			<0.001
T1	34 (4.11%)	31 (1.63%)	
T2	247 (29.87%)	378 (19.93%)	
T3	374 (45.22%)	1040 (54.82%)	
T4	172 (20.80%)	448 (23.62%)	
N-stage			<0.001
N0	542 (65.54%)	1061 (55.93%)	
N1	202 (24.43%)	391 (20.61%)	
N2	83 (10.04%)	445 (23.46%)	
MMR			0.105
dMMR	26 (3.14%)	85 (4.48%)	
pMMR	801 (96.86%)	1812 (95.52%)	
Tumor size (cm)			<0.001
<4	431 (52.12%)	579 (30.52%)	
>=4	396 (47.88%)	1318 (69.48%)	
CEA (ug/L)			<0.001
<3	530 (64.09%)	1086 (57.25%)	
>=3	297 (35.91%)	811 (42.75%)	
CA19-9 (U/mL)			0.016
<37	554 (66.99%)	1179 (62.15%)	
>=37	273 (33.01%)	718 (37.85%)	
Neutrophil (×10^9^/L)			0.064
<1.80	32 (3.87%)	54 (2.85%)	
1.80-6.30	763 (92.26%)	1736 (91.51%)	
>6.30	32 (3.87%)	107 (5.64%)	
Lymphocyte (×10^9^/L)			0.158
<1.10	69 (8.34%)	124 (6.54%)	
1.10-3.20	733 (88.63%)	1701 (89.67%)	
>3.20	25 (3.02%)	72 (3.80%)	
Hb (g/L)			<0.001
<115	71 (8.59%)	230 (12.12%)	
115-150	483 (58.40%)	1154 (60.83%)	
>150	273 (33.01%)	513 (27.04%)	
PLT (×10^9^/L)			0.036
<125	22 (2.66%)	45 (2.37%)	
125-350	743 (89.84%)	1650 (86.98%)	
>350	62 (7.50%)	202 (10.65%)	

### 3.3 Nomogram construction and model validation

**Figure 2:**
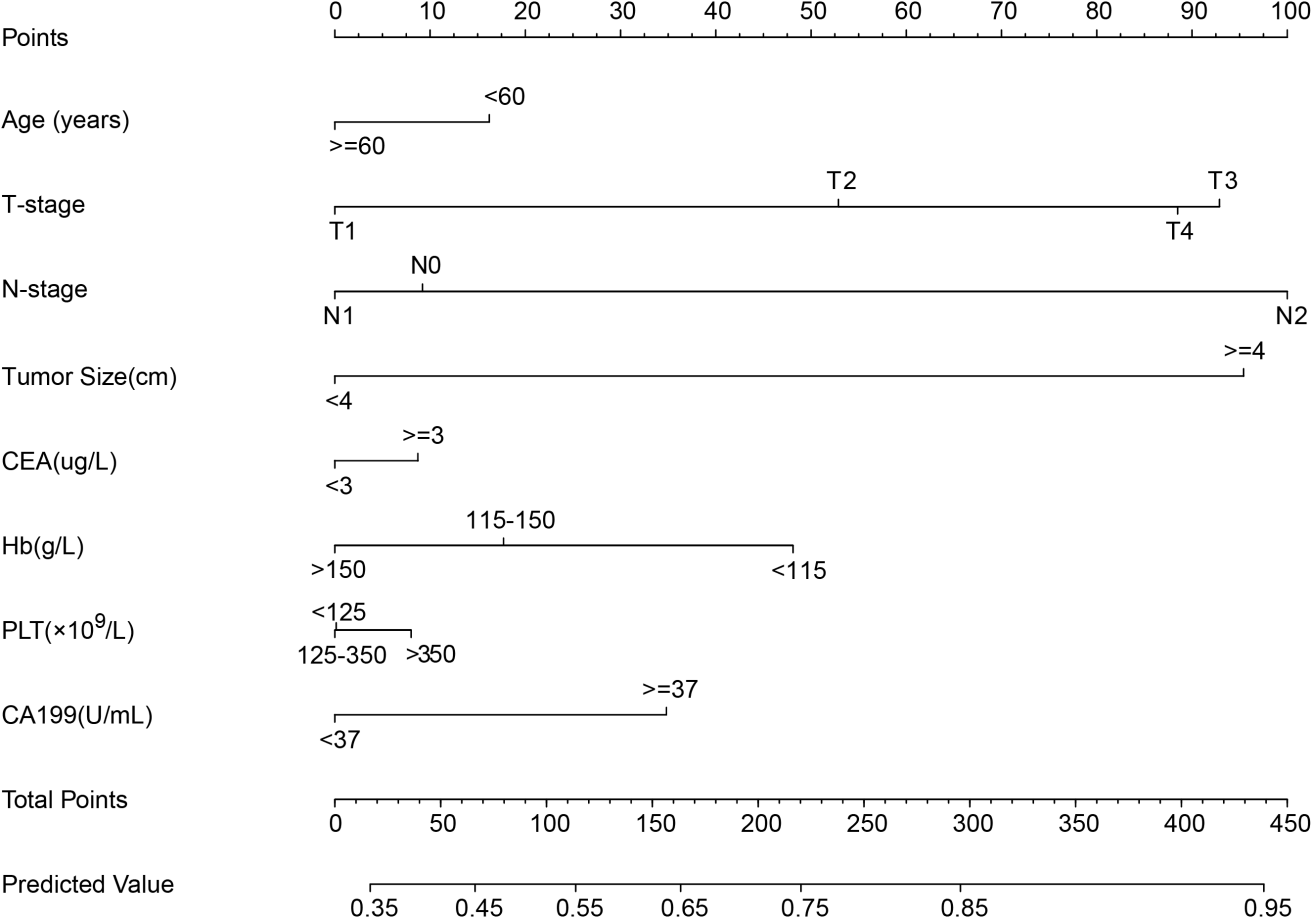
Nomogram for predicting the probability of detecting 12 LNs in RC patients.

**Figure 3:**
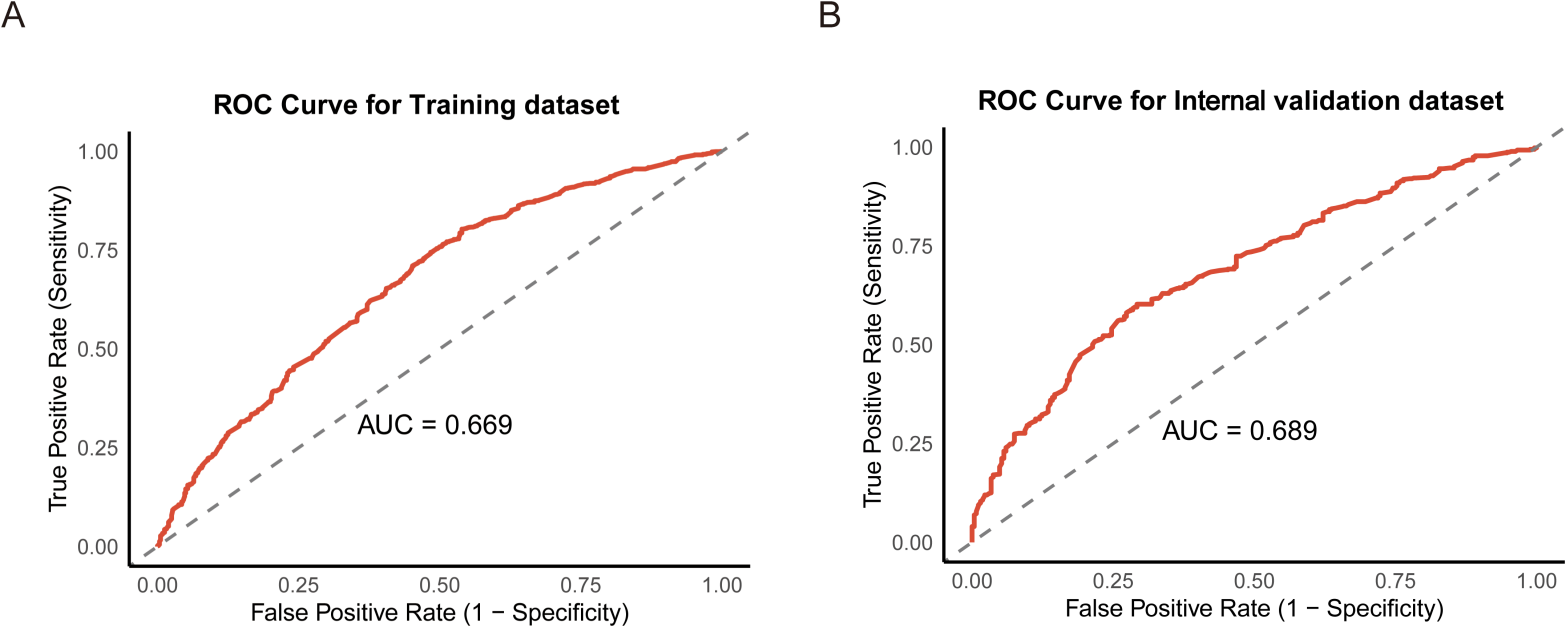
Receiver operating characteristic (ROC) curves for the training **(A)** and internal validation datasets **(B)**.

**Figure 4:**
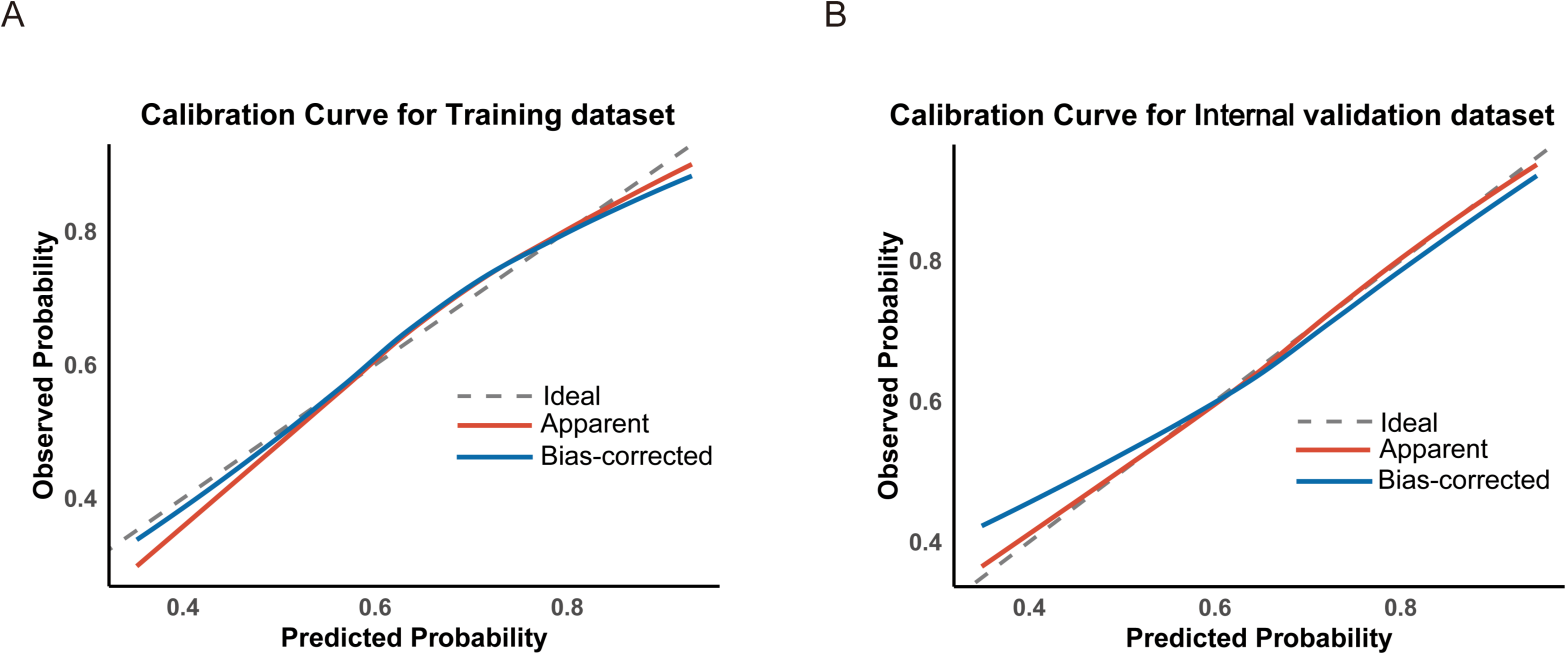
Calibration curves assessing the agreement between predicted and observed probabilities in the training **(A)** and internal validation **(B)** datasets.

**Figure 5:**
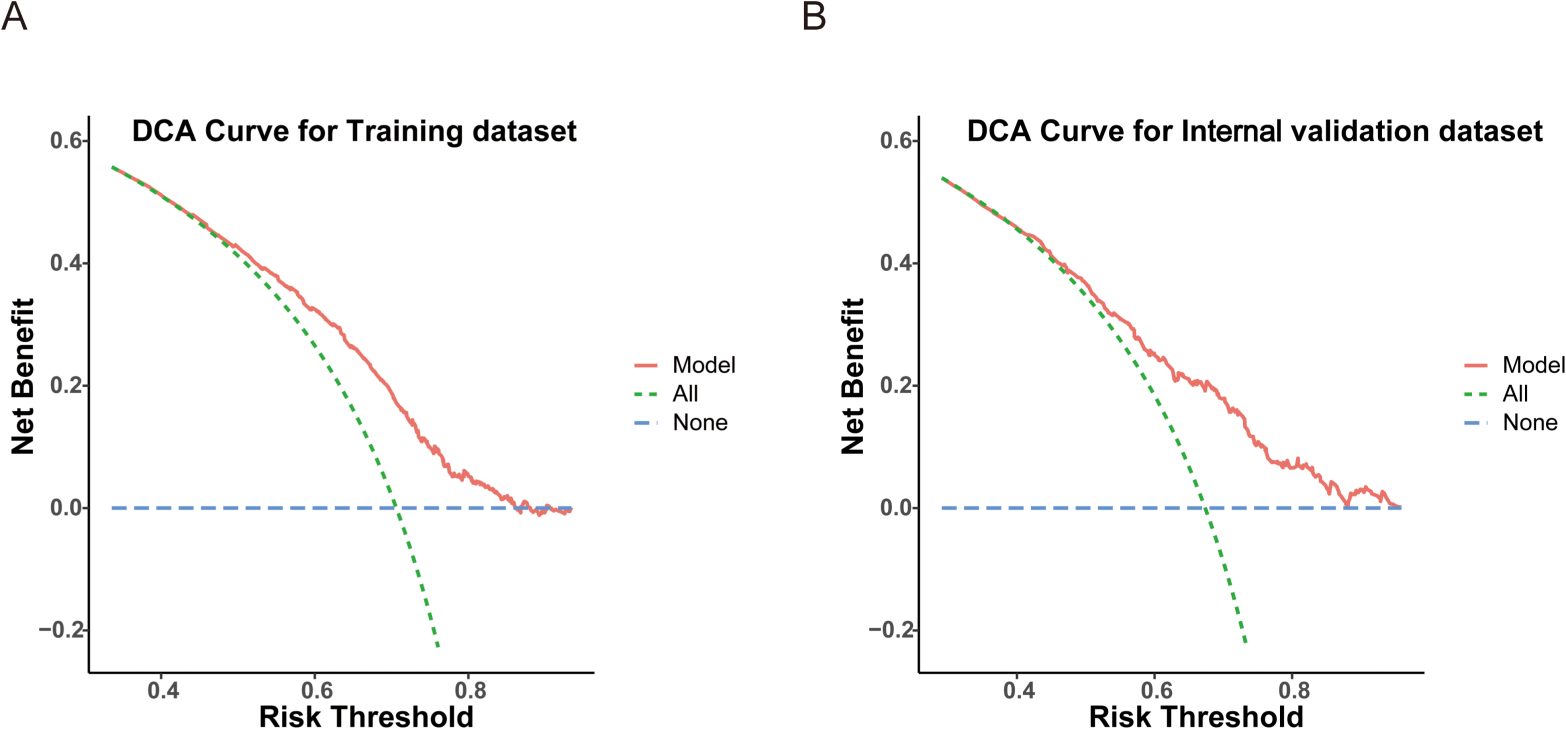
Decision curve analysis (DCA) evaluating the clinical utility of the model in the training **(A)** and internal validation **(B)** datasets.

Based on the variables identified as significantly associated with retrieval of ≥12 LNs, a nomogram was developed to provide a visual tool for individualized prediction of adequate LN retrieval (**Figure 2**). The predictive performance of the nomogram was assessed using receiver operating characteristic (ROC) curve analysis. The area under the curve (AUC) was 0.669 in the training cohort and 0.689 in the internal validation cohort (**Figure 3**), demonstrating moderate discrimination ability of the model. Calibration plots were generated to evaluate the agreement between the predicted probabilities by the nomogram and the actual observed outcomes. Both the training and internal validation cohorts showed good concordance, with calibration curves closely following the ideal diagonal line (**Figure 4**). Furthermore, decision curve analysis (DCA) was performed to assess the clinical utility of the nomogram. The results showed that across a broad range of threshold probabilities, the nomogram provided a higher net benefit compared to either the treat-all or treat-none strategies (**Figure 5**), indicating its potential value in guiding clinical decision-making.

## 4 Discussion

This retrospective study identified several key factors potentially influencing the likelihood of retrieving 12 LNs after RC surgery. These factors include age, T stage, N stage, CEA, CA19-9, tumor size, Hb level, and PLT. Based on these variables, we developed a predictive model and visualized it as a nomogram. The ROC curve, calibration curve, and DCA indicated that the model demonstrated good accuracy and strong clinical utility.

Previous studies on the prediction of LN yield are limited. Through a retrospective analysis of 1,375 RC patients, Zhang et al. found that age, tumor size, and CEA level were associated with the number of LNs retrieved (14), in alignment with our results. Similarly, Emile et al. conducted a retrospective analysis of 67,529 CRC patients and concluded that higher age and neoadjuvant chemoradiotherapy (nCRT) were major contributors to inadequate LN retrieval; in contrast, higher TNM stage, higher tumor grade, and minimally invasive surgery were associated with the successful retrieval of 12 LNs (15). In the current study, we also found that higher T and N stages were significant predictors of adequate LN yield. Retrieving at least 12 LNs has become a standard criterion for assessing postoperative treatment plans in CRC. However, patients who undergo nCRT may experience a reduced number of detectable LNs. Yang et al. retrospectively analyzed 257 RC patients who underwent laparoscopic radical resection after nCRT and found that retrieving fewer than 12 LNs did not negatively impact long-term survival (16). A similar conclusion was reached by Ryu and colleagues (17). Our current study lacks detailed preoperative treatment information, limiting the applicability of our model in patients who received nCRT. We recommend that clinicians individualize treatment strategies for these patients.

In the nomogram results, patients with elevated CA19–9 levels and low Hb levels scored higher in nomogram, suggesting these factors significantly influence LN yield. Patients with elevated CA19–9 levels were more likely to have ≥ 12 LNs retrieved than patients with normal or low CA19–9 levels. This implies that CA19–9 may not only reflect tumor biology, it may also be associated with lymphatic changes within the tumor microenvironment. Previous studies have linked elevated CA19–9 levels with tumor progression, local invasion, and distant metastasis (18–20). LNs are also critically involved in immune and anti-tumor responses (21, 22). For example, in a retrospective analysis of 2,244 stage III colon cancer patients, Kuo et al. found that a higher number of negative LNs correlated with better prognosis (23). Similarly, Quan et al.’s study on pN1 right-sided colon cancer showed improved DFS and OS in patients with more than nine negative LNs (24), emphasizing the importance of LNs. Tumor cells can interact with surrounding stromal and inflammatory cells to form an inflammatory tumor microenvironment (25). Engle et al. showed that overexpression of CA19–9 in mice accelerated pancreatitis, and this inflammatory response was reversed by anti-CA19–9 antibodies, indicating a close relationship between CA19–9 and inflammation (26). Zhang et al. performed single-cell sequencing on three CA19-9-positive and three CA19-9-negative patients and found that cancer-associated fibroblasts produced CA19–9 to promote the M2 polarization of macrophages in the pancreatic tumor microenvironment, thereby accelerating tumor progression (27). These pro-inflammatory effects and microenvironmental changes induced by CA19–9 may attract more immune cell infiltration and LN response, resulting in an increased number of retrievable LNs postoperatively.

In the present study, we found that patients with below-normal Hb levels had significantly higher rates of retrieving ≥ 12 LNs, suggesting that anemia may affect LN detection. In a case-control study involving 5,075 early-onset CRC patients, Fritz et al. found that anemia was associated with increased CRC risk (28). One potential cause of anemia is iron deficiency, which has been shown to impair cancer immune surveillance and alter the tumor immune microenvironment, facilitating tumor progression (29). These anemia-induced changes may lead to reactive hyperplasia of mesenteric LNs, increasing their size and making them more identifiable in surgical specimens. Additionally, anemia is closely linked with hypoxia. Guo et al. found that genetic variants in hypoxia-related genes were associated with increased CRC risk and enhanced immune infiltration (30), possibly leading to the promotion of LN immune response and indirectly increasing the number of detectable LNs. However, current research on the relationship between anemia and LN yield remains limited, and the underlying mechanisms are not fully understood. Further basic and clinical studies are needed to validate these findings.

This was a single-center retrospective study based on clinical data from RC patients. We developed a nomogram with good predictive performance and practical value, providing a useful tool for assessing the likelihood of retrieving 12 LNs postoperatively. Using this model, patients with fewer than 12 LNs detected postoperatively may not necessarily be classified as high-risk candidates requiring adjuvant chemotherapy. Pathologists can preliminarily estimate whether the number of retrieved LNs meets the threshold of 12 using this model. When the model predicts fewer than 12 LNs, it can also help reduce the tissue sampling time and facilitate more efficient staging assessment in the pathology department. However, this study has several limitations, including a relatively small sample size and potential selection bias. AUC values suggest moderate discriminatory power, factors such as surgeon experience, pathologist proficiency, and neoadjuvant chemoradiotherapy, which were not included in the analysis, may have influence outcomes. Moreover, the model has not been externally validated. Future studies involving larger, multi-center, or prospective cohorts are warranted to externally validate and optimize the model, thereby improving its robustness and clinical applicability.

## 5 Conclusion

In conclusion, we developed an effective model for predicting the likelihood of retrieving 12 LNs after RC surgery, providing valuable guidance for clinicians in staging and treatment planning. This model not only facilitates more accurate prognostic assessment, it also contributes to improving the quality of surgical and pathological evaluation, ultimately aiding in the optimization of individualized patient management. In the future, an online calculator or application based on the nomogram may be developed to facilitate easy and accessible use in real-world clinical settings.

## Data Availability

The raw data supporting the conclusions of this article will be made available by the authors, without undue reservation.
